# Chinese Herbal Products for Female Infertility in Taiwan: A Population-Based Cohort Study

**DOI:** 10.1097/MD.0000000000003075

**Published:** 2016-03-18

**Authors:** Yu-Chiang Hung, Chao-Wei Kao, Che-Chen Lin, Yen-Nung Liao, Bei-Yu Wu, I-Ling Hung, Wen-Long Hu

**Affiliations:** From the Department of Chinese Medicine (Y-CH, C-WK, Y-NL, B-YW, I-LH, W-LH), Kaohsiung Chang Gung Memorial Hospital and School of Traditional Chinese Medicine, Chang Gung University College of Medicine; School of Chinese Medicine for Post Baccalaureate (Y-CH), I-Shou University, Kaohsiung; Management Office for Health Data (C-CL), China Medical University Hospital, Taichung; Fooyin University College of Nursing (W-LH); and Kaohsiung Medical University College of Medicine (W-LH), Kaohsiung, Taiwan.

## Abstract

Female infertility and low birth rate are significant public health issues with profound social, psychological, and economic consequences. Some infertile women resort to conventional, complementary, or alternative therapies to conceive. The aim of this study was to identify the Chinese herbal products (CHPs) most commonly used for female infertility in Taiwan.

The usage of traditional Chinese medicine (TCM) and the frequency of CHP prescriptions to infertile women were determined based on a nationwide 1-million randomly sampled cohort of National Health Insurance Research Database beneficiaries. Descriptive statistics and multiple logistic regression analysis were employed to estimate the adjusted odds ratio (aOR) for TCM usage and potential risk factors.

In total, 8766 women with newly diagnosed infertility were included in this study. Of those, 8430 (96.17%) had sought TCM treatment in addition to visiting the gynecologist. We noted that female infertility patients with risk factors (e.g., endometriosis, uterine fibroids, or irregular menstrual cycle) were more likely to use TCM than those without TCM medication (aOR = 1.83, 1.87, and 1.79, respectively). The most commonly used formula and single CHP were Dang-Gui-Sha-Yao-San (17.25%) and Semen Cuscutae (27.40%), respectively. CHP formula combinations (e.g., Dang-Gui-Sha-Yao-San plus Wen-Jing-Tang 3.10%) or single Chinese herbal combinations (e.g., Semen Cuscutae plus *Leonurus japonicus* 6.31%) were also commonly used to treat female infertility. Further well-conducted, double-blind, randomized, placebo-controlled studies will be needed to evaluate the efficacy and safety of these CHP combinations for female infertility.

## INTRODUCTION

**FIGURE 1:**
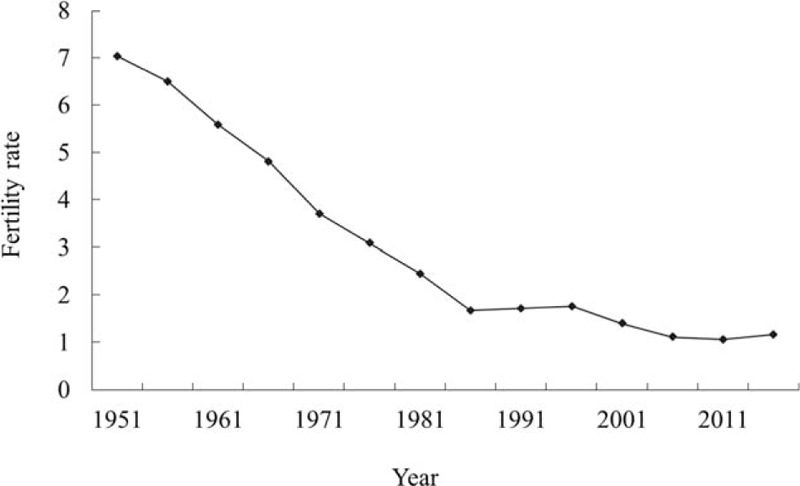
Fertility rate of women of childbearing age in Taiwan (1951–2014).^2^.

A low fertility rate results in a low birth rate and an aging country. For the purpose of this study, we focus on female fertility. The prevalence of infertility has increased since 1990. There was about 48.5 million infertile couples all over the world in 2010.^1^ The female total fertility rate fell from 7.04 in 1951 to 1.165 in 2014 on Taiwan (Figure 1).^2^ Taiwan has thus become one of the countries with the lowest fertility rates in the world. Female fertility has been defined as “a paradoxical phenomenon of power between the biological and psychosexual self.”^3^ On the other hand, female infertility is defined as the inability of a woman within childbearing age to conceive despite having frequent, unprotected intercourse for at least 1 year.^4^ Infertility affects about 10% to 20% of couples trying to achieve pregnancy in many industrialized countries.^1,5^ There are an increasing number of couples seeking medical treatment.^6^ The prevalence of 1-year and 2-year infertility among newly married couples is 12.5% and 6.6%, respectively, in China.^7^ Female fertility decreases gradually with age, and more rapidly so after the age of 37.^8,9^ A previous study has estimated that more than 186 million ever-married women of reproductive age in developing countries are infertile.^10^ Some researchers have identified infertility as an under-observed, yet significant public health issue with profound social, psychological, and economic consequences.^11–13^

The main causes of female infertility are ovulatory disorders, endometriosis, pelvic adhesion, tubal blockage and other tubal abnormalities, hyperprolactinemia, and congenital (septate uterus) and acquired (myomas and synechiae) uterine abnormalities.^14,15^ Conventional therapies for female infertility include clomiphene citrate, human menopausal gonadotropin, follicle-stimulating hormone, human chorionic gonadotropin, gonadotropin-releasing hormone analogs, aromatase inhibitor, and metformin.^16,17^ However, some fertility drugs increase the risk of cancer^18–22^ or have no pharmacological effect in infertile women aged 40 years or older.^8,23^ Complementary or alternative therapies such as traditional Chinese medicine (TCM) may be indicated in such cases.

Along with Western medicine, TCM is the most common form of medicine used in Taiwan. Chinese products and herbs are the most popular components of TCM. Approximately 30% of all patients use TCM in Taiwan, and about 90% of TCM users receive Chinese herbal products (CHPs) as treatment for various diseases.^24–26^ A previous meta-analysis has reported that Chinese herbal medicine combined with clomiphene citrate significantly increases the pregnancy and ovulation rates, improves the cervical mucus score, and reduces the miscarriage rate compared with clomiphene citrate alone.^27,28^ However, large-scale, extensive studies of the most commonly used Chinese products and herbs for female infertility are lacking.

Infertility diagnoses and treatments, including those of traditional Chinese and Western medicines, are reimbursed by the National Health Insurance (NHI) in Taiwan. All medication is prescribed electronically, and the information is stored in a computer database by the National Health Research Institute (NHRI) to form the National Health Insurance Research Database (NHIRD). Due to the high coverage by the NHI, which included up to 98.3% of the population by the end of 2006 and counting,^29^ nearly all CHPs prescribed for female infertility are included in the NHIRD, making it feasible to conduct a nationwide analysis on the management of female infertility using this database.

The aim of this study was to identify the CHPs most commonly used for female infertility by analyzing a nationwide database in Taiwan. The results of this study can be used as a practical reference for further clinical trials or pharmacological experiments.

## METHODS

### Data Source

Taiwan's NHI is a governmental nationwide initiative launched in 1995. The system is compulsory for Taiwanese citizens, making the coverage rate very high (it was nearly 98% of 23 million Taiwanese citizens in 1998). The government of Taiwan appointed the NHRI to lead and manage a project that would establish a database of all NHI claims. The database was named NHIRD.

This study used the Longitudinal Health Insurance Database (LHID), which is a subset of NHIRD. The LHID randomly selected 1 million insured people from 1996 to 2000. The database includes the registry for beneficiaries, the diagnosis records (based on the International Classification of Diseases, 9th Revision, Clinical Modification [ICD-9-CM]), drug prescriptions, and other medical services; the data were updated every year until 2011. LHID and NHIRD are similar in terms of age and sex distribution. The NHRI encoded the patients’ personal information for privacy protection and provided researchers with anonymous identification numbers prior to research. This study was approved by the Institutional Review Board of China Medical University in central Taiwan (CMU-REC-101-012).

### Study Population

**FIGURE 2:**
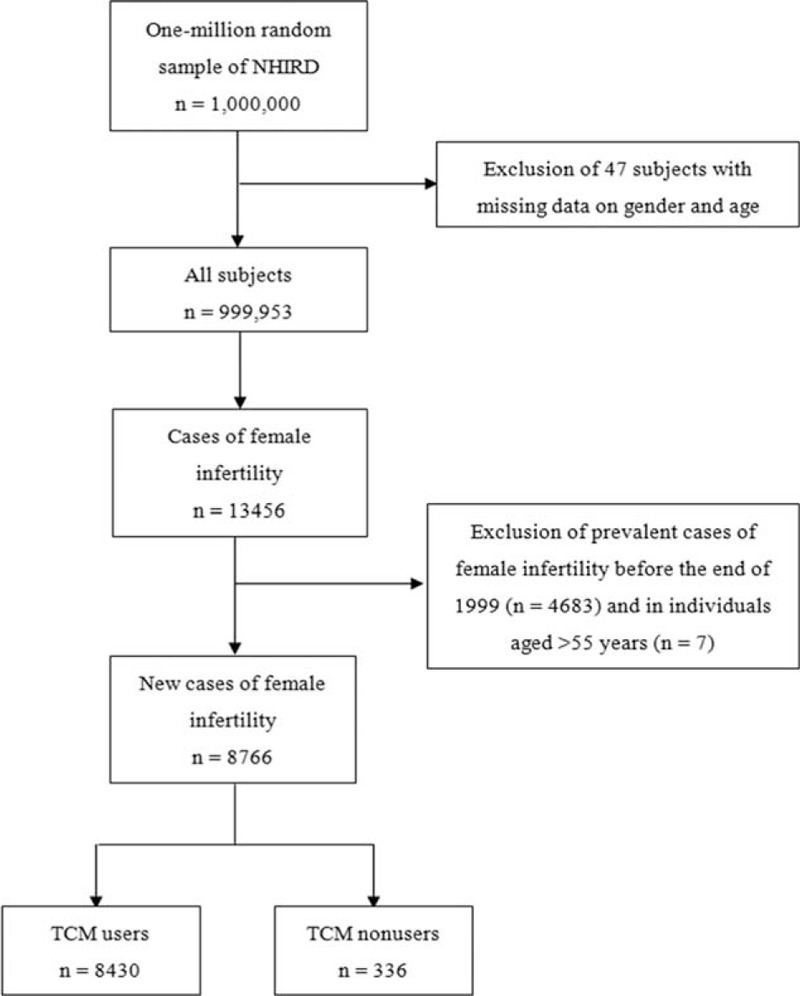
Flowchart of recruitment of subjects from the 1-million random sample of the National Health Insurance Research Database (NHIRD) from 2000 to 2011 in Taiwan. TCM = Traditional Chinese medicine.

We selected the study subjects from LHID. Figure 2 shows the flowchart of study population selection. Of the initial 1 million individuals, we excluded 47 subjects because of missing data on age and sex. Next, we restricted the population to the cases of female infertility (ICD-9-CM 606 and 628) in the LHID (n= 13,456). After exclusion of prevalent cases of female infertility before the end of 1999 (n = 4683) and subjects aged >55 years (n = 7), we finally included 8766 new-onset female infertility patients in our study population. TCM user was defined as an individual who resorted to TCM treatment at least once before the end of the LHID recruitment period. TCM nonuser was defined as an individual who had never received TCM treatment.

We selected some demographic factors and comorbidities to determine the potential risk factors associated with TCM usage in infertile women. The comorbidities were defined from inpatient and outpatient diagnoses made prior to the diagnosis of infertility. The comorbidities included polycystic ovary syndrome (ICD-9-CM 256.4), endometriosis (ICD-9-CM 617), uterine fibroids (ICD-9-CM 218), and irregular menstrual cycle (ICD-9-CM 626.4). We also collected data on herbal formulas and single herbal products prescribed by TCM doctors to infertile women.

### Statistical Analysis

For studying the outcomes of CHPs for female infertility, descriptive statistical data of TCM users and nonusers are presented as the mean and standard deviation (SD) for age, and as the number and percentage for age group and comorbidities. To determine the association between TCM usage and potential risk factors, the odds ratio (OR) and 95% confidence interval (CI) were estimated by multivariable logistic regression. Adjusted OR would be mutually adjusted for potential source of bias. We also present the frequency and percentage of top 10 CHPs. A significance level of *α* *=* 0.05 was used. Data management and analysis were performed by using SAS 9.4 software (SAS Institute Inc, Cary, NC).

## RESULTS

Of the 8766 female infertility patients, 8430 were TCM users and 336 were TCM nonusers (Table 1). There were no differences in the distribution of age and polycystic ovary syndrome between TCM users and nonusers. The women with endometriosis, uterine fibroids, or irregular menstrual cycle were more likely to use TCM than those without TCM medication (aOR = 1.83, 1.87, and 1.79, respectively).

**TABLE 1:**
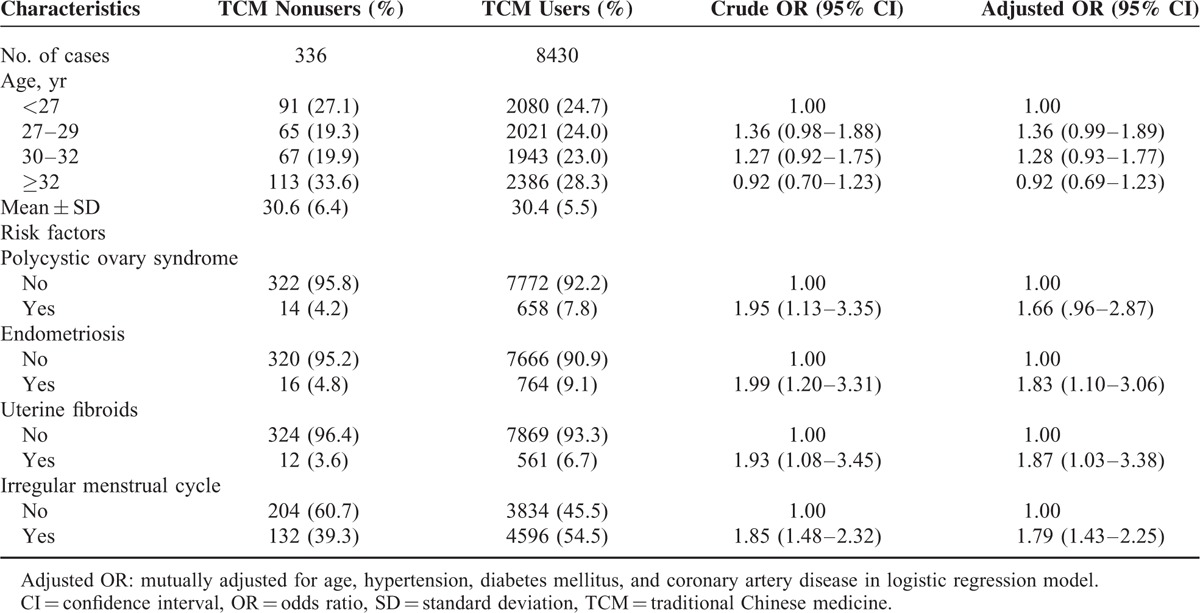
Demographic Characteristics and Results of Multiple Logistic Regression Showing the Adjusted Odds Ratio and 95% Confidence Interval of Patients With Female Infertility From 2000 to 2011 in Taiwan

**FIGURE 3:**
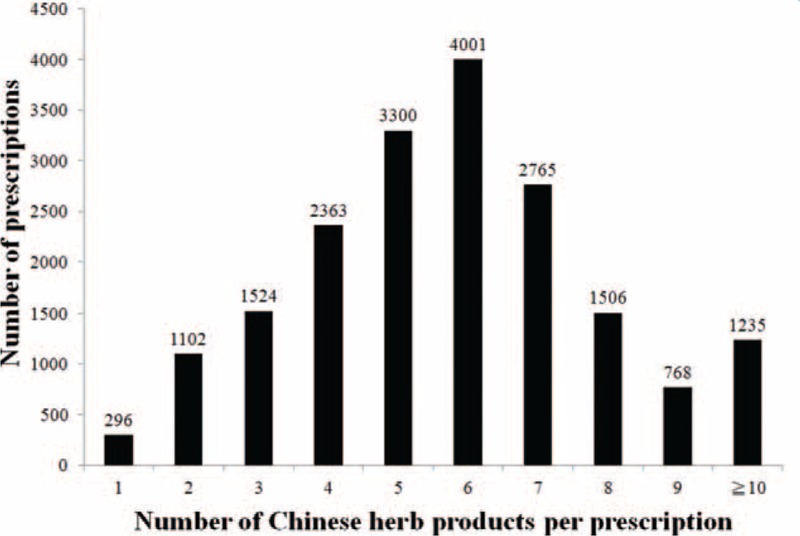
Distribution of the number Chinese herbal products per prescription.

Figure 3 shows the distribution of the number of CHPs per prescription. Ninety-eight percent of all prescriptions for female infertility contained at least 2 CHPs per prescription. There was an average of 5.81 Chinese herbs in a single prescription. Table 2 shows the top 10 CHP formulas prescribed by TCM doctors to treat female infertility. Dang-Gui-Sha-Yao-San was the most commonly prescribed herbal formula (17.25% of 18,860 prescriptions), followed by Wen-Jing-Tang (16.35%), Jia-Wei-Xiao-Yao-San (14.85%), Zou-Gui-Wan (14.57%), and You-Gui-Wan (9.97%). Semen Cuscutae was the most commonly prescribed single CHP (27.40%) (Table 3), followed by *Leonurus japonicus* (13.55%), *Fructus ligustri lucidi* (13.43), *Cyperus rotundus* L. (12.13%), and *Dipsacus asper* Wall (11.71%).

**TABLE 2:**
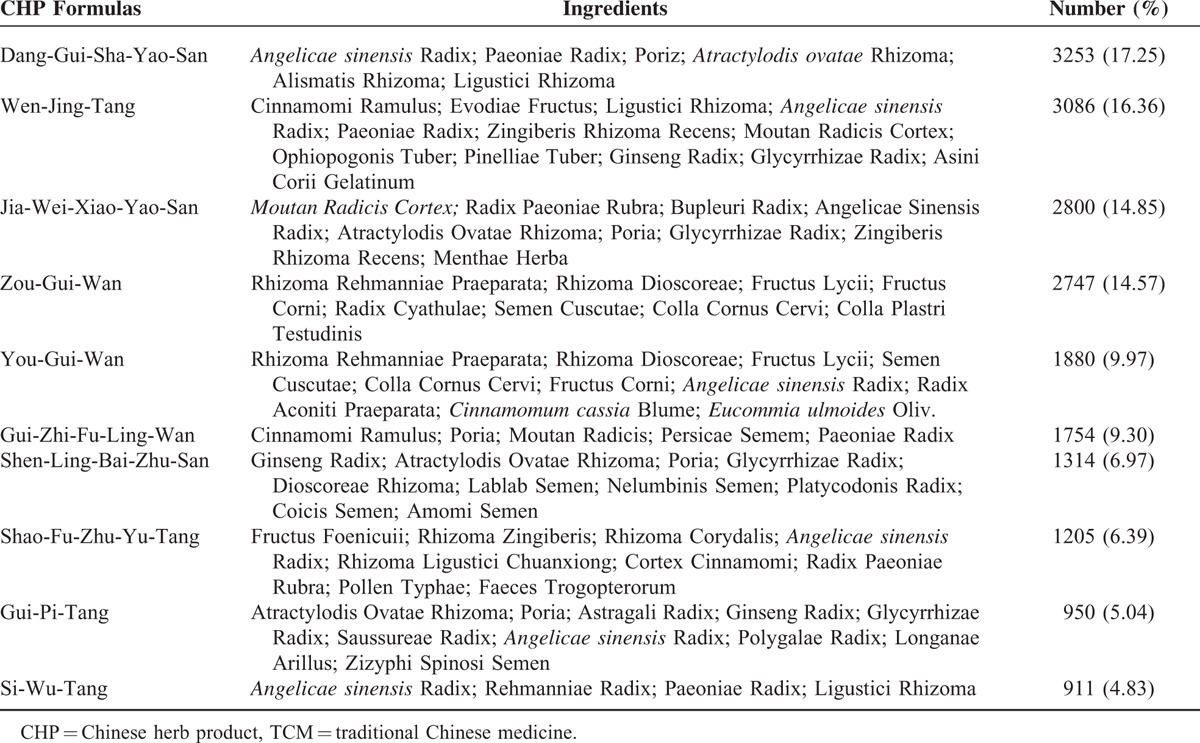
Top 10 Chinese Herbal Product Formulas Prescribed by Traditional Chinese Medicine Doctors to Female Infertility Patients From 2000 to 2011 in Taiwan (Total Number of Prescriptions, n = 18,860)

**TABLE 3:**
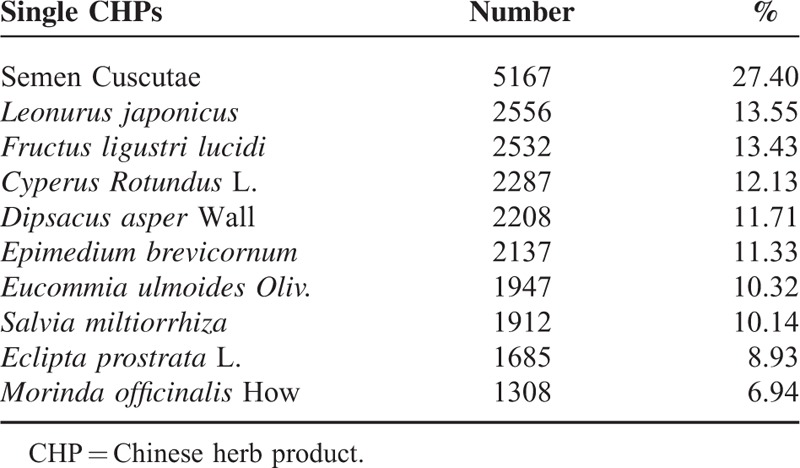
Top 10 Single Chinese Herbal Products Prescribed by Traditional Chinese Medicine Doctors to Female Infertility Patients From 2000 to 2011 in Taiwan (Total Number of Prescriptions, n = 18,860)

Table 4 shows the top 5 most used combinations of CHPs pairs. Dang- Gui-Sha-Yao-San plus Wen-Jing-Tang was the most commonly prescribed 2-formula combination (3.1%). *Fructus ligustri lucidi* plus *Eclipta prostrata* L. was the most commonly prescribed 2-single CHP combination (6.31%).

**TABLE 4:**
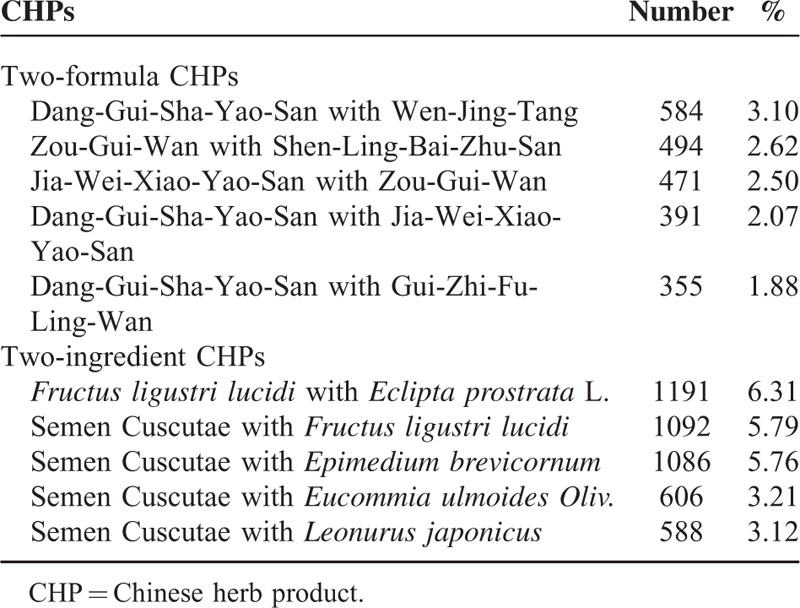
Top 5 Most Prescribed Chinese Herbal Product Pair Combinations to Female Infertility Patients in Taiwan From 2000 to 2011 (Total Number of Prescriptions, n = 18,860)

## DISCUSSION

This is an important large-scale survey of Chinese herbal prescriptions and herbs used in the treatment of female infertility in a Taiwanese population. We found that, in addition to visiting the gynecologist, the majority of these patients (96.17%) also sought TCM treatment. It seemed high demand for infertile women with traditional Chinese medicine treatment as a complementary or alternative medicine. We explored the correlation of use of TCM treatment, and noted that female infertility patients with infertility risk factors such as endometriosis, uterine fibroids, or irregular menstrual cycle were more likely to use TCM than those without TCM medication. Endometriosis, uterine fibroids, or irregular menstrual cycle may affect infertility. Our study has shown that the more comorbidities per disease, the more likely are individuals to seek treatment—either traditional Chinese or Western medicine treatment.

We found that the most common individual Chinese herbal prescriptions and herbs among female infertility patients were Dang-Gui-Sha-Yao-San and *Semen Cuscutae*. Dang-Gui-Sha-Yao-San is a herbal mixture used for abdominal pain during pregnancy. Previous reports revealed that Dang-Gui-Sha-Yao-San corrects luteal phase insufficiency^30,31^ via an antioxidant mechanism^32^ or an antagonistic action on both prostaglandin F2-α and acetylcholine-induced uterine contraction.^33^*Semen Cuscutae* improves ovarian endocrine dysfunction and increases estrogen receptor expression in the hippocampus, hypothalamus, and pituitary glands, as well as luteinizing hormone receptor expression in the ovaries.^34^ One other study reported that *Semen Cuscutae* regulates the proliferation and apoptosis of the decidua and cytotrophoblasts, thus preventing spontaneous abortions.^35^ In Taiwan, Dang-Gui-Sha-Yao-San and *Semen Cuscutae* often used syngergistically to optimize the treatment for female infertility through individualized therapy. Another herbal formula, Wen-Jing-Tang, improves the endocrine condition in the treatment of disturbances of ovulation^36^ and suppresses the contraction of uterus smooth muscle.^37^*Leonurus japonicus* was reported to have an antioxidative effect on the uterus.^38^

In addition, we also noted that combinations of CHPs such as Dang-Gui-Sha-Yao- San plus Wen-Jing-Tang or single herbal combinations such as *Semen Cuscutae* plus *Leonurus japonicus* were commonly used to treat female infertility. Mixed formulations are usually prescribed in combination to enhance efficacy, minimize toxicity, or tailor the treatment according to individual needs. Our results also showed that on average, 5.81 types of CHPs are prescribed per prescription. These results should be taken into account by physicians when devising individualized therapy for female infertility.

Since the NHIRD is a very complete database including 22.60 million of 22.96 million people, the representativeness of this database is reliable and strong to provide some information of the patients’ medical seeking behavior and physicians’ prescription patterns in Taiwan. The completeness of NHIRD and nationwide population-based study design increase the validity of the study results. However, 1 limitation of our study was that we did not determine the efficacy of therapeutic effects. We found the most commonly used CHPs, but not necessarily the most effective ones. Although the NHIRD contains large amounts of prescription data, chart-level records (i.e., physician notes, laboratory reports, and imaging studies) are not available. Thus, it was not possible to evaluate the effectiveness of the treatments. The other one was that the database did not contain some potential confounders such as education, smoking, alcohol consumption, and economic state, which may also be associated with female infertility.

In conclusion, CHPs are commonly used for the treatment of female infertility in Taiwan. Various CHPs with particular effects are used synergistically to optimize the treatment of female infertility. Data mining analysis helped identify the most commonly prescribed CHP combinations. Dang-Gui-Sha-Yao-San and *Semen Cuscutae* are the most frequently prescribed CHPs by TCM doctors in Taiwan for female infertility. These results provide information for individualized therapy of female infertility. Randomized, double-blind control trials will be needed to evaluate the effectiveness of TCM on female infertility in the future.
